# A Bi-CMOS electronic photonic integrated circuit quantum light detector

**DOI:** 10.1126/sciadv.adk6890

**Published:** 2024-05-17

**Authors:** Joel F. Tasker, Jonathan Frazer, Giacomo Ferranti, Jonathan C. F. Matthews

**Affiliations:** Quantum Engineering Technology Labs, H. H. Wills Physics Laboratory, University of Bristol, Bristol BS8 1TL, UK.

## Abstract

Complimentary metal-oxide semiconductor (CMOS) integration of quantum technology provides a route to manufacture at volume, simplify assembly, reduce footprint, and increase performance. Quantum noise–limited homodyne detectors have applications across quantum technologies, and they comprise photonics and electronics. Here, we report a quantum noise–limited monolithic electronic-photonic integrated homodyne detector, with a footprint of 80 micrometers by 220 micrometers, fabricated in a 250-nanometer lithography bipolar CMOS process. We measure a 15.3-gigahertz 3-decibel bandwidth with a maximum shot noise clearance of 12 decibels and shot noise clearance out to 26.5 gigahertz, when measured with a 9–decibel-milliwatt power local oscillator. This performance is enabled by monolithic electronic-photonic integration, which goes below the capacitance limits of devices made up of separate integrated chips or discrete components. It exceeds the bandwidth of quantum detectors with macroscopic electronic interconnects, including wire and flip chip bonding. This demonstrates electronic-photonic integration enhancing quantum photonic device performance.

## Teaser

Integrating electronics and photonics into one silicon chip simultaneously speeds up and shrinks detectors for quantum technology.

## INTRODUCTION

Photonic integrated circuits (PICs) are a compelling approach to developing quantum technology (*1*, *2*), and they underpin proposed architectures for optical quantum computing (*3*, *4*). Complimentary metal-oxide semiconductor (CMOS)–compatible PIC platforms, such as silicon on insulator (SOI) photonics (*5*, *6*), offer paths to scaling up the manufacture of photonic devices for quantum technology in commercial foundries. This may prove critical in the construction of universal quantum computers because the scale and performance required of components to build quantum computers are beyond anything yet constructed in information technologies (*3*).

Since initial experiments with silicon quantum photonic circuits (*7*, *8*), CMOS compatibility for electronic-photonic integration has been a clear goal for quantum photonics. This is because it would enable integration at scale of components generating and using quantum states of light with the required high-performance classical readout and control electronics. However, to date, the development of foundry fabricated electronic PIC (ePIC) platforms (*6*, *9*) has been driven by the performance demands of classical applications, with demonstrations including direct detection receivers (56 gigabit/s) (*10*) and coherent receivers (128 gigabit/s) (*11*) for fiber optics telecommunciations and coherent detector arrays with active pixel amplifiers for three-dimensional (3D) imaging (*12*). High-bandwidth ePIC detector technology has not been developed to meet the necessary conditions of high-performance quantum technology.

**Fig. 1.**
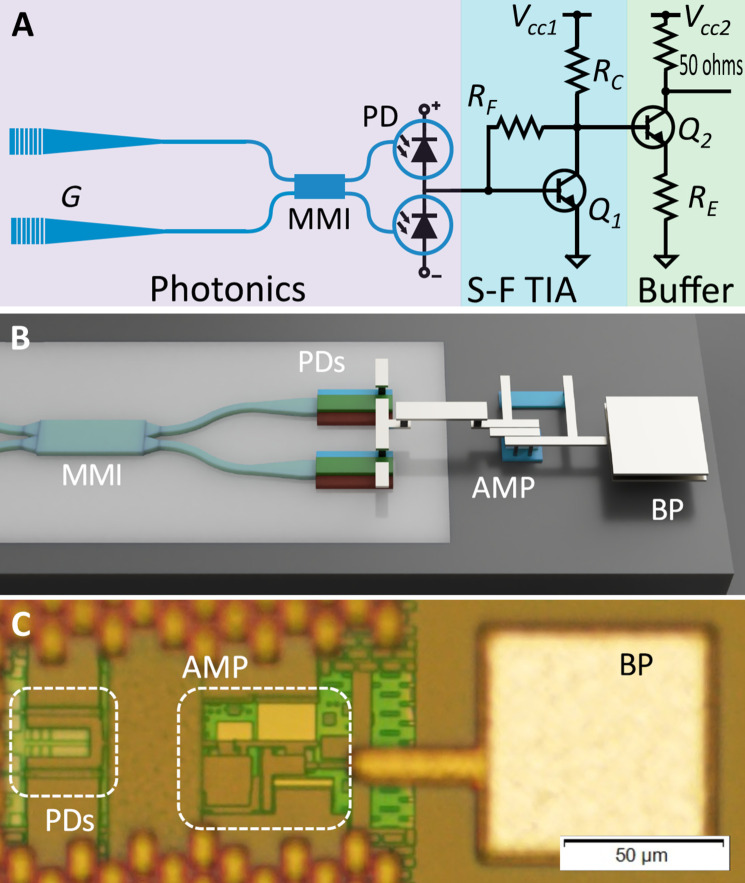
A bipolar CMOS integrated homodyne detector for measuring quantum light. (**A**) The detector schematic. The photonics include grating couplers (*G*), mode converters, strip waveguides, a MMI beam splitter, and germanium-silicon photodiodes (PDs). The electronics are a two-stage TIA design. The first transistor (*Q*_1_) forms a common-emitter shunt-feedback TIA; the second (Q_2_) constitutes a 50-ohm output buffer amplifier. *R*_F_, *R*_C_, and *R*_E_ label the feedback, load, and emitter resistors, respectively. BP labels the detector bondpad. (**B**) A 3D illustration of connections between components using three of the five metal layers in the SG25H5_EPIC process (*9*) used to fabricate the device. Light gray indicates SOI, and dark gray indicates bulk silicon. (**C**) A microscope image of the detector illustrates scale. AMP labels the TIA and buffer amplifier stages. This device fits within an 80-μm by 220-μm footprint.

Here, we demonstrate that electronic-photonic integration can be applied to enhance quantum technologies. We report integration in one monolithic ePIC chip (Fig. 1) of all the electronics and silicon photonics needed for homodyne detection of quantum optical signatures (*13*). The detector has a measured 3-dB bandwidth of 15.3 ± 0.1 GHz and a maximum measured shot noise clearance of 12 dB. We measure more than 5 dB of shot noise clearance at 26.5 GHz that is the maximum frequency measurable with our current analysis equipment.

Homodyne detectors suitable for quantum applications can measure weak signals down to the single-photon level by interfering them with a local oscillator at an optical beam splitter. The gain and noise specifications provided by classical coherent receivers are unsuitable for this type of detection, and so the design of specialized amplification electronics is required to realize homodyne detectors suitable for quantum application. The resulting interference is observed in the subtraction of photocurrents from a pair of photodiodes placed at the two beam splitter outputs. This subtraction current requires amplification, and when the amplification electronics are of low enough noise, the homodyne detector is sensitive enough to reveal quantum noise signatures in the input. This is quantified by the clearance between optical shot noise and the electronic noise of the detector, which is sufficiently measured using a coherent laser source as the local oscillator input into one mode of the beam splitter and vacuum into the remaining mode (*14*). Quantum technology applications of homodyne detectors include squeezed light–enhanced gravitational wave detection (*15*, *16*), quantum state tomography (*13*), measuring continuous-variable cluster states (*17*, *18*) for quantum computing, and continuous-variable quantum communication (*19*, *20*).

Waveguide-integrated beam splitters have been used for homodyne detection with silica-on-silicon (*21*) and lithium niobate PICs (*22*). In silicon-on-insulator photonics, on-chip germanium p-i-n photodiodes have been integrated with waveguides and interfaced with discrete amplifier electronics for quantum random number generation and coherent state tomography (*23*) and as a chip-scale receiver for continuous-variable quantum key distribution (*24*). In these cases, the detector bandwidths were limited respectively to ~100 and ~10 MHz by discrete electronics, mounted on printed circuit boards (PCBs). Consequently, microelectronic amplifiers were wirebonded to silicon PICs, and the resulting detectors demonstrated 3-dB bandwidths of 1.7 GHz (*25*) and 1.5 GHz (*26*)—these detectors were respectively used to measure squeezing over a 9-GHz bandwidth and observe shot noise clearance out to 20 GHz. Subsequently, this approach has been used for quantum random number generation (100 gigabit/s) (*27*) and as a receiver for 10-GBaud continuous-variable quantum key distribution (*28*).

Two-chip modules interfacing electronic chips with photonics chips, such as (*25*, *26*), use lithographic fabrication of each chip. This approach assists high-volume manufacture when combined with automated assembly to interface the two chips. However, multichip homodyne detectors will remain fundamentally limited in the bandwidth over which they can detect quantum light. This is because a remaining limiting factor in the speed of these detectors is the 20- to 100-fF capacitance overhead of the electrical bondpad interconnection (*29*), which interfaces the PIC with the integrated electronics. Flip chip interfaces introduce similar capacitance overheads and, so, also restrict the possible bandwidth of hybrid integration using macroscopic interconnects. These capacitance overheads are especially damaging to the bandwidth of transimpedance amplifiers (TIAs), particularly where high gain is required for shot noise clearance (*14*). This is why integrated photodiodes can readily achieve O(10) GHz (*30*) when connected to an external 50-ohm load for classical applications, whereas homodyne detectors have been limited to units of gigahertz (*25*). This, in turn, limits the clock rate of continuous-variable quantum computing (*4*) and photonic neuromorphic computing (*31*) that use electronic-photonic homodyne detection. We overcome this quantum noise–limited bandwidth limitation using monolithic integration of electronics and PICs.

## RESULTS

The reported single-chip homodyne detector is illustrated in Fig. 1. Our design simultaneously satisfies the gain and noise specifications required to achieve tens of gigahertz of bandwidth with shot noise clearance for quantum technology application, while using ePIC technology. This provides a route for scalable fabrication and minimization of each detector’s footprint to O(100) μm^2^ and attains quantum noise–limited speed performance not achievable before with standard homodyne detection. Here, ePIC technology removes all bondpad and packaging parasitics, with connections between photonics and electronics made in the metal interconnect layers of the back end of line. A 3D model and a microscope image of the detector are shown in Fig. 1, B and C.

The properties of a homodyne detector suitable for measuring quantum states of light for technology can be characterized and verified completely by a laser, as shown in, e.g., (*14*). This includes measurements of responsivity, common mode rejection ratio (CMRR), shot noise clearance, and scaling of optical shot noise with local oscillator power. We characterize the bandwidth, CMRR, linearity, and responsivity of the device. Light is coupled into the chip using grating couplers, and multimode interferometers (MMIs) are used as beam splitters. A continuous-wave tuneable laser (PurePhotonics PPCL550) at 1550 nm and amplified with an erbium-doped fiber amplifier (PriTel) is used as a local oscillator (LO). A variable optical attenuator (Oz Optics) adjusts LO power. Note that a coherent laser source input into one port of the beam splitter, with vacuum in the other, is sufficient to perform a complete characterization of the detector’s ability to measure quantum states of light (*14*). Noise measurements are recorded using a Keysight N9020B MXA electronic spectrum analyzer (ESA) with a 26.5-GHz bandwidth. The detector output is amplified before the ESA with an external SHF810 broadband amplifier to increase the detector output above the ESA noise floor. Photodiode and transistor biases are supplied from source meters (Keysight U2722A and Keithley 2450) that are also used to monitor the two individual photocurrents of the diodes. We compare measured photocurrents when injecting LO at the top and bottom MMI ports, finding a splitting ratio of 42:58 transmission to reflection. This imbalance results in a net photocurrent at the amplifier input and excess electronic noise at the amplifier output (see the Supplementary Materials). We offset this effect by reducing the bias on the bottom photodiode (as appears in Fig. 1) until the photocurrents are matched, with a maximum difference of 80 μA at the maximum LO power. This reduces the quantum efficiency of the bottom diode to 72% of its maximum value.

The top and bottom photodiodes are each reverse-biased at 2.6 and −0.3 V, respectively, relative to an amplifier input voltage of 0.9 V.The transistor supplies, *V*_cc1_ and *V*_cc2_ (see Fig. 1A), are set to 2.2 and 1.65 V, respectively. To account for signal distortion from PCB transmission lines, coaxial cables, and the external amplifier, we measure S21 parameters using a Keysight N5225A network analyzer of a transmission line test structure and the other components used in the experiment.

**Fig. 2.**
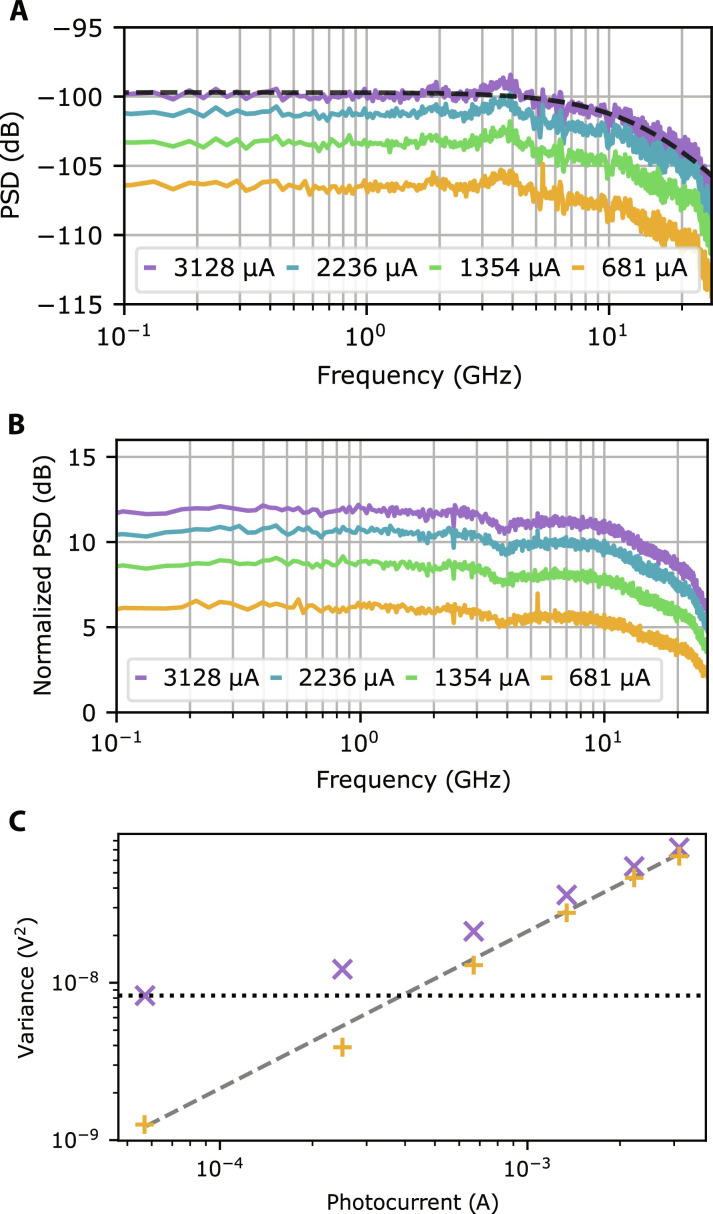
Homodyne detector characterization. (**A**) Power spectral density (PSD) of the detector where ESA noise and the amplifier dark noise have been subtracted in addition to cable, amplifier, and transmission line corrections. The legend represents the total photocurrent measured on both photodiodes. The dashed line shows a fit to Eq. 3 and gives a 3-dB bandwidth of 15.3 ± 0.1 GHz. (**B**) Power spectral density of the detector normalized to the amplifier electronic noise. Both plots have been corrected to remove cable losses and electronic noise. (**C**) Raw and electronic noise subtracted detector noise variance at 1 GHz against total photocurrent. The horizontal dotted line represents the electronic noise level. A linear fit to the data (dashed line) indicates a gradient of 0.99 ± 0.01, demonstrating the presence of vacuum shot noise up to a maximum clearance of 12 dB.

We perform a bandwidth measurement by optimizing coupling at maximum power using the monitored photocurrent and then recording a series of spectra on the ESA as the variable optical attenuator adjusts the input power from 9 to −26.5 dBm. We also record the ESA displayed average noise level (the intrinsic ESA noise) for later subtraction from the data. All spectra are recorded at 100-kHz resolution bandwidth (RBW) over a 26.5-GHz span. The results of this are plotted in Fig. 2. By fitting the detector response to a second-order Butterworth response, we obtain a 3-dB bandwidth of 15.3 ± 0.1 GHz. The 3-dB bandwidth is the figure of merit typically used in quantum technology to define homodyne detector speed performance. However, because the clearance of the detector extends beyond the bandwidth of our ESA, we expect that the presented detector can readily be used to measure quantum signatures over a much broader bandwidth. We include in the Supplementary Materials the analysis of the presented detector’s expected shot noise clearance beyond 100 GHz, using Eq. 2—this can be tested with higher bandwidth analysis equipment. We fit the clearance of the detector with *A*/(*B* + *Cf*^2^) + 1, where *A* describes the optical shot noise, *B* is the white noise terms of Eq. 2, and *C* is the latter frequency-dependent terms (see the Supplementary Materials). The fit suggests that the shot noise clearance extends far beyond the measured bandwidth, vanishing beyond 100 GHz. In practice, we anticipate the photodiode transit time bandwidth to become limiting (*32*).

Grating coupler losses are measured using grating-to-grating test structures that we included on the ePIC chip. This yields an average of ~4.0 dB per coupler. We characterize the photodiode responsivity by comparing the sum of measured photocurrents to off-chip LO power and correcting for grating coupler losses. From this, we obtain a maximum photodiode responsivity of 0.47 A/W at 2-V bias, which decreases to 0.34 A/W on the bottom photodiode (as appears in Fig. 1) when the bias is decreased to compensate for subtraction imbalance. The resulting global efficiency for on-chip quantum states, including optical loss, photodetector responsivity, and shot noise clearance, is 29%. These values include MMI insertion loss, which could not be measured individually because of the monolithic nature of the device.

**Fig. 3.**
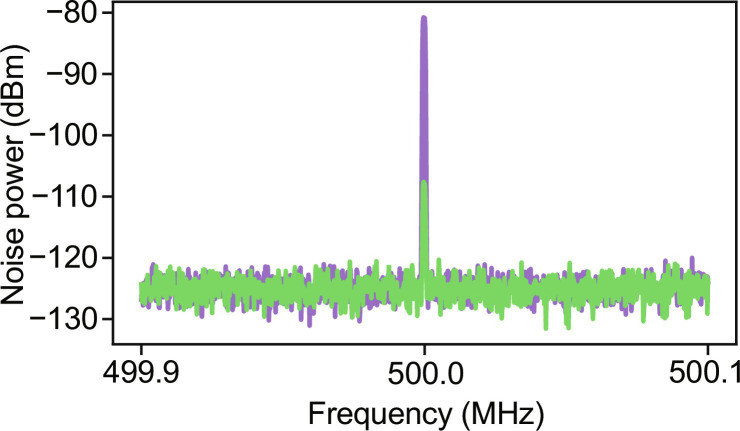
Detector CMRR at 500 MHz. The LO power is set to generate 10 μA of total photocurrent and the noise power recorded with one or both photodiodes reverse-biased. We observe a maximum of 27-dB CMRR at 500 MHz.

CMRR measurements are made by intensity-modulating the LO using an electro-optic modulator and comparing the signal with either both photodiodes biased as above or one biased and the other disconnected to eliminate its photocurrent contribution. The ESA is set to 10-kHz RBW and a span ±0.1% of the modulation frequency. We observe a CMRR of 27 dB at 500 MHz (Fig. 3), which is limited by the intrinsic splitting ratio of our MMI. In future devices, this value can be improved by substituting the current static MMIs with thermoelectric tunable Mach-Zehnder interferometers (*25*) or equivalently through iterative optimization of MMI splitting ratio. The introduction of tunable phase shifters and iterative MMI optimization in the photonic side of the device would not imply any modification to our amplification electronics and therefore not compromise the detector’s quantum noise–limited bandwidth.

## DISCUSSION

An ePIC quantum light detector is reported, combining photonics and transimpedance amplification electronics for readout within an 80-μm by 220-μm footprint. This was achieved because of the CMOS compatibility of silicon photonics, which can benefit the scalability and manufacturability of photonic quantum information processors and could be a necessity when considering the stringent timing limits imposed by feed-forward and delay lines (*33*). Our ePIC detector design brings together standard electronic and photonic components available in a commercially available ePIC process—this further exemplifies the power of the accessibility of multiproject wafer services (*2*), which can accelerate application of ePIC homodyne detectors for quantum technology. The reported device’s performance was achieved with the first attempt at a fabrication run. Future study with appropriate resources would investigate the yield of fabricating the presented design and future improvements. The detector’s 15.3 ± 0.1–GHz 3-dB bandwidth is an order of magnitude greater than the previous fastest demonstrations and surpasses the speed performance limits of homodyne detectors constructed from macroscopic wirebond interconnects (*25*). It has been shown that an optical parametric amplifier (OPA) per spatial mode measured can be used to increase tolerance to loss, with 43-GHz bandwidth measurement of 5 dB of squeezing (*34*). Our work shows that comparable speed performance can be achieved without the need of additional OPAs using ePIC technology that can implement each high-speed detector within a small physical footprint of O(100) μm^2^. Future work could explore the combination of ePIC homodyne detectors with PIC OPAs to increase tolerance to loss. With complexity of silicon quantum photonics continuing to increase (*35*), ePIC homdodyne detectors can be a route to adding on chip capability. However, two-photon absorption occurring in silicon at telecommunication wavelengths can be problematic when high-intensity light is guided within waveguides, for example, in state generation (*36*), because it exposes quantum states to nonlinear loss. Hybrid integration of silicon nitride waveguides with silicon waveguides (*37*) can provide a route to integrate nonlinear light sources with ePIC homodyne detectors.

The detector’s global efficiency of 29% for on-chip quantum states necessitates future effort to improve device efficiency. For example, the current device efficiency would correspond to direct detection of 0.68 dB of squeezing for an input state with 3 dB of squeezing and direct detection of 1.43 dB of squeezing for an input state with 15 dB (*38*) of squeezing. The current device’s upper limit of directly measurable squeezing is 1.49 dB. The reported detector reaches a shot noise clearance of 12 dB, which equates to a shot noise contribution of the total homodyne detector efficiency of at least η_SNC_ = 94%. This is as given by $\eta_{\text{SNC}}=1-\sigma_{\text{EN}}^{2}/\sigma_{0}^{2}$ , where $\sigma_{\text{EN}}^{2}$ is the variance of the raw output of the detector and $\sigma_{0}^{2}$ is the variance of the electronic technical noise contribution. Higher gains, and thus higher shot noise efficiencies, will be possible in future devices through multistage amplifier designs without sacrificing bandwidth (*39*).

The dominating factor in the present device though is photodiode responsivity. First, we sacrificed responsivity in one diode due to unequal splitting ratio in the detector’s beam splitter—as shown previously [for example, on chip in (*25*)], tunable beam splitters are one route to immediately increase the effectively lower responsivity from 0.34 to 0.47A/W. This would correspond to an efficiency improvement to 37%. Higher–quantum efficiency homodyne detectors can be achieved by optimizing the design of the photonic components and incorporating greater efficiency components that have already been demonstrated separately in classical applications. For example, in a study that designed, modeled, fabricated, and tested silicon-germanium waveguide pin photodiodes integrated with SOI waveguides over a range of device length and reverse bias voltages, responsivities of up to 1.19 A/W (95% quantum efficiency) were reported with 30-GHz bandwidths (*40*). These devices were fabricated with 200-mm SOI wafers and standard CMOS processes on a fully integrated photonic platform in CEA-Leti’s cleanroom facilities (*40*). We identify this as a path to increasing device efficiency to >90% in the near term and beyond. To further increase the utility of ePIC homodyne detectors as fiber-coupled devices, such as for hybrid use with other materials, fiber-coupling efficiencies of 95% have been observed with edge couplers (*41*). Incorporating these improvements will enable ePIC detectors to reach the efficiency performance requirements of future quantum technologies.

We believe that the current detector’s footprint and speed performance already open the application of ePIC homodyne detectors to miniaturized and high-speed receivers for quantum communications (*19*, *24*), higher–clock rate cluster state characterization (*17*, *18*) and large arrays of coherent receivers for continuous-variable photonic quantum computing (*4*) and photonic neural networks operating below the Landauer limit (*31*). Beyond detectors, we anticipate future applications of ePICs to increase the performance of quantum device control, including increasing the number of simultaneously controlled phase shift parameters beyond O(10^2^) in highly programmable quantum devices (*42*). We expect that the combination of miniaturized readout and control within ePICs will reduce the requirements on optical delay lines for quantum technologies using state measurement and feedforward (*33*). This is important for large-scale implementations of quantum technology including multiplexed sources of quantum states (*43*), quantum state engineering (*44*), and measurement-based and time-multiplexed quantum computing (*4*, *45*).

## MATERIALS AND METHODS

### Chip design and fabrication

The chip was designed and characterized in-house with fabrication outsourced to the Leibniz Institute for High Performance Microelectronics (IHP). We chose IHP’s SG25H5_EPIC process, which features a 250-μm silicon node, germanium-based photodiodes with *f*_3dB_ > 60 GHz and vertically integrated heterojunction bipolar transistors (HBTs) for radio frequency (RF) applications using 250-nm lithography with a specified transition frequency *f*_T_ = 220 GHz and a breakdown voltage of 1.7 V (*9*). The RF performance of these HBTs is comparable to the lateral n-channel metal oxide semiconductor field-effect transistors (NMOS) in (*46*–*48*). This is due to the vertical carrier transport of the HBT, meaning that speed is less dependent on the lithography resolution allowing vertical bipolar transistors to outperform NMOS devices at the same process node (*49*). The HBTs are integrated in the same front-end-of-line process as the silicon-on-insulator waveguides and active optical components, such as modulators and photodiodes.

The IHP fabrication process begins with an SOI wafer optimized for photonics, with a 220-nm silicon layer thickness and a 2-μm-thick buried oxide layer. A “local-SOI” approach is used in which SOI regions that are to be used for bipolar CMOS devices are etched down to the silicon substrate. Bulk silicon is selectively regrown epitaxially in these regions and is subsequently planarized using chemical-mechanical planarization. Patterning of electronic and photonic structures is conducted in parallel, and the electrical contacts to the photodiodes and transistors are made with the same process step (*50*). Devices are then connected through a single shared back end of line with five metal layers.

A 3D model and a microscope image of the detector are shown in Fig. 1, B and C. A 20-μm trace connects the photodiode subtraction signal to the amplifier input. Our parasitic extraction simulations estimate the parasitic capacitance of this interface at 7 fF, compared with 105 fF when simulating a single bondpad at the amplifier input. The ePIC is bonded to a purpose-made PCB designed for high-frequency operation. Vertical silicon capacitors (Murata UWSC, 1 nF) are used on the PCB for power supply decoupling on transistor and photodiode biases. The ePIC itself contains additional vertical metal-insulator-metal capacitors located next to each component for additional supply filtering. The wirebond at the output of the TIA is kept short to minimize parasitic inductance, in keeping with typical RF packaging best practices. This limits any unwanted filtering at the amplifier output, although we emphasize that this is a minor effect relative to parasitic capacitance at the amplifier input.

### Transimpedance amplifier

The TIA used consists of an HBT common-emitter amplifier in shunt-feedback configuration, followed by a 50-ohm buffer amplifier for interfacing with standard RF test equipment (see Fig. 1). The bandwidth of a single-stage shunt-feedback TIA with an ideal second-order Butterworth response is given by (*39*)

$$f_{3\text{dB}}=\sqrt{\frac{A_{0}\;f_{A}}{2\pi C_{\text{in}}R_{F}}}$$ (1)

where *C*_in_ is the total capacitance at the amplifier input, *R*_F_ is the feedback resistance, and *A*_0_*f*_A_ is the gain-bandwidth product (*f*_A_ is the pole frequency and *A*_0_ is the DC gain) (*39*). A monolithic design reduces *C*_in_ by minimizing the stray capacitance between the photodiodes and amplifier due to bondpads or other wiring related sources. This comes in addition to the already low capacitance associated with integrated photodiodes and high-performance HBTs—integrated photodiodes with capacitances as low as 9 fF and amplifier input capacitance of order of 100 fF have been reported (*30*, *51*). This is in stark contrast to the packaging and layout-associated parasitic capacitance on a PCB of up to tens of picofarads (*23*, *52*). Equation 1 demonstrates the fundamental trade-off between the detector bandwidth and the transimpedance gain from the subtraction photocurrent to output voltage. Larger transimpedance gains are desirable to ensure that the detector noise lies above the noise floor of any subsequent equipment and provide the maximum shot noise clearance when an LO field is applied. However, the practically usable *R*_F_ and achievable bandwidth are constrained by the total input capacitance and the gain-bandwidth product. In the case of a single transistor amplifier, the gain-bandwidth product is proportional to the transistor transition frequency, *f*_T_ via *A*_0_*f*_A_ ≈ *C*_I_/*C*_L_*f*_T_, where *C*_I_/*C*_L_ is ratio of transistor input and load capacitances (*53*).

The input-referred current noise power spectral density is given by

In,TIA2(f)=4kBTRF+2qICβ+2qIC(2πCT)2gm2f2+4kTRb(4πCPD)2f2 (2)

where *I*_C_ is the HBT collector current, β is the DC current gain, *g*_m_ is the transistor transconductance, and *R*_b_ is the base resistance (*53*). The first two terms are white-noise terms, specifically the feedback resistor Johnson noise and base current shot noise, respectively. The latter terms scale quadratically with frequency to a limit set by the photodiode junction capacitance and total capacitance, including parasitics, presented to the amplifier input.

The amplifier is implemented with two n-p-n transistors as shown in Fig. 1A, the design of which is provided as part of the SG25H5_EPIC process development kit (PDK). The transition frequency *f*_T_ is maximized for a particular collector current density—for our collector area, this corresponds to an optimal bias current *I*_C_ of 4.5 mA. Achieving the optimal collector current requires careful tuning of the biasing resistors *R*_C_ and *R*_E_ for a given transimpedance gain *R*_F_. We perform lumped element SPICE (Simulation Program with Integrated Circuit Emphasis) simulations of the amplifier to optimise resistances with *V*_cc1_ = 2.2 V and *V*_cc2_ = 1.7 V dictated by the transistor breakdown voltage. The chosen resistors are *R*_F_ = 600 ohms, *R*_c_ = 250 ohms, and *R*_E_ = 35 ohms where the feedback resistance has been chosen to provide sufficient clearance above the fundamental thermal noise floor of the 50-ohm termination resistor in RF test equipment. Photonic layout was performed using IPKISS and Cadence Virtuoso. Simulations, electronic design, layout, and postlayout electronic simulations were performed using Cadence Virtuoso using PDK SPICE models provided by IHP.

The gain spectrum of an ideal shunt-feedback TIA is that of a second-order Butterworth filter, given by

$$G\left(f\right)=\frac{A_{0}^{2}}{1+{\left(\frac{i2\pi f}{i2\pi f_{3\text{dB}}}\right)}^{2}}$$ (3)

where $A_{0}^{2}$ is the absolute gain at zero frequency. The second-stage buffer operates as a unit gain amplifier and can be assumed to have a bandwidth approximately equal to the transistor transition frequency (*53*).
