# Supplementary material for: A Bi-CMOS electronic photonic integrated circuit quantum light detector
Source: Sci Adv. 2024 May 17;10(20):eadk6890. doi: 10.1126/sciadv.adk6890 (PMC11100555; doi:10.1126/sciadv.adk6890)
Supplement: Supplementary file 1 — Supplementary Text Figs. S1 to S4 [file sciadv.adk6890_sm.pdf]

Science Advances
AAAS

Supplementary Materials for

A Bi-CMOS electronic photonic integrated circuit quantum light detector

Joel F. Tasker *et al.*

Corresponding author: Jonathan C. F. Matthews, jonathan.matthews@bristol.ac.uk

Sci. Adv. **10**, eadk6890 (2024)
DOI: 10.1126/sciadv.adk6890

This PDF file includes:

Supplementary Text
Figs. S1 to S4

Supplementary Text

Current offset excess noise

Due to the MMI imbalance before the photodiodes, we observe a power dependent net current offset, i_{diff}, at the amplifier input. This results in excess electronic noise at the amplifier output which we attribute to a combination of LO relative intensity noise and the amplifier's DC current dependent gain. Fig. S1 shows characterisation of the detector with symmetric 2 V reverse biases on each photodiode.

Reducing quantum efficiency with lowered bias voltage

The response of a photodiode to incident light is linear up to its saturation point. Beyond which the increase in photocurrent rolls off, although does not vanish. This saturation point depends strongly on the reverse bias. In the two-photodiode homodyne arrangement, maintaining a smaller bias on one photodiode effectively limits that photodiode efficiency at higher powers without sacrificing linearity at lower powers. While precise balancing across a range of powers would require tuning the bias voltage for each power, in practice setting the bias at maximum LO power upper bounds the net photocurrent, and hence the classical laser noise contribution in the homodyne detector.

Shot noise clearance limit

The clearance of a balanced homodyne detector is described by a function of the form,

$$\mathrm{SNC} = \frac{A}{B + Cf^2} + 1$$

where A describes the shot noise contribution and B and C represent the white noise and quadratic noise terms of Eq. 2 in the main text (*52*). We fit this function to the measured clearance data, plotting the data and fit in Fig. S2.

Raw power spectral density

For completeness, we plot in Fig. S3 the raw PSD output from the spectrum analyser with zero correction. It includes the electronic noise of the detector~(0 μA). The plotted data is a composition of the spectrum analyser noise and various other frequency dependent noises and losses that lower frequency quantum noise limited homodyne detectors, and detectors with higher gains, do not contend with.

Noise linearity across the measured spectrum

Here we demonstrate that our PSD has linear dependence with the local oscillator power across the entire spectrum measurable with our current analysis equipment. In Fig.~S4A, the electronic noise of the detector is subtracted from the acquired PSD traces, which are then normalised to the trace of lowest non-zero LO power. The height of each normalised trace matches the corresponding photocurrent ratio, showing that the PSD depends linearly on the input LO power. This is further evidenced in Fig.~S4B, which plots the slope obtained from a linear fit of the power dependence of the PSD in 200 equally sized bins across each measured spectrum. We obtain an average slope of 1.00±0.03.

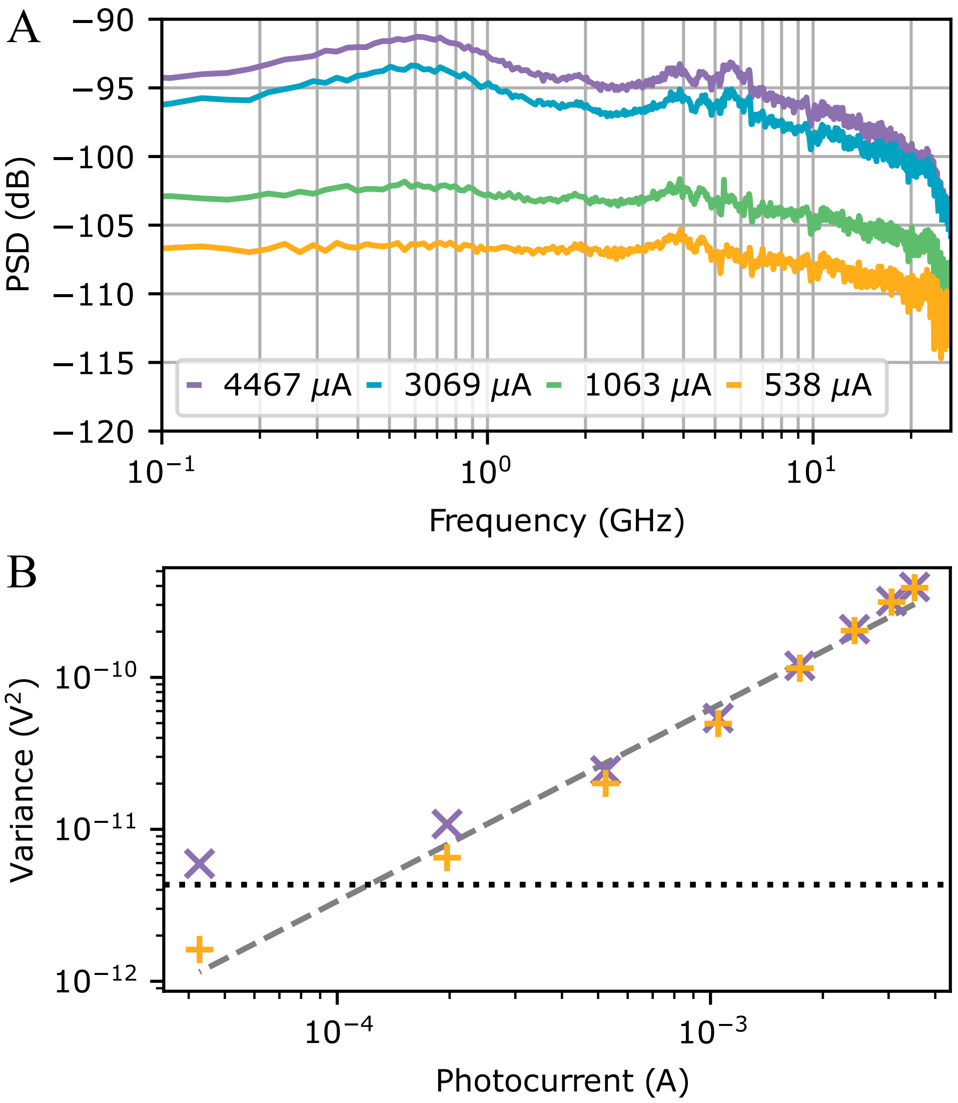

Fig. S1. Characterisation with imbalanced photocurrents. A, Power spectral density (PSD) of the device response at different LO powers. ESA DANL, amplifier electronic noise and cable/ PCB transmission losses have been removed. We attribute the excess noise centred at 6 GHz to intensity noise from the EDFA. B, Raw (purple crosses) and electronic noise subtracted (orange pluses) noise variances against total photocurrent. Dashed lines indicate the electronic noise level and a linear fit to the data, respectively. The fit gives a gradient of 1.26 ± 0.01, indicating the presence of excess classical noise in addition to vacuum shot noise.

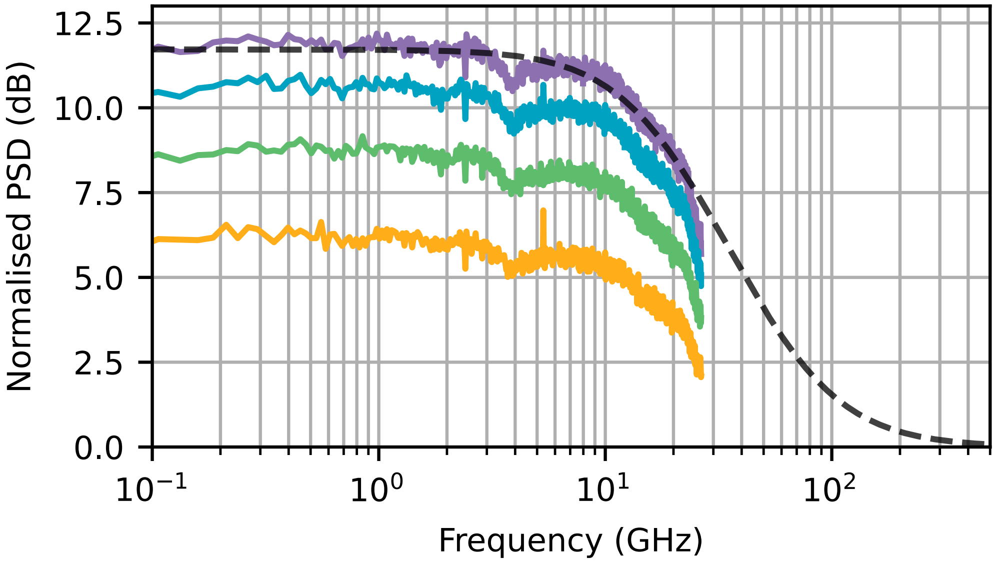

Fig. S2. Shot noise clearance fit. We normalise the measured detector shot-noise response to the amplifier and spectrum analyser electronic noise to obtain the ratio of quantum to classical noise, or clearance. We extrapolate the trend beyond our 26.5 GHz measurement bandwidth through a fit to Equation 1, suggesting clearance beyond 100 GHz.

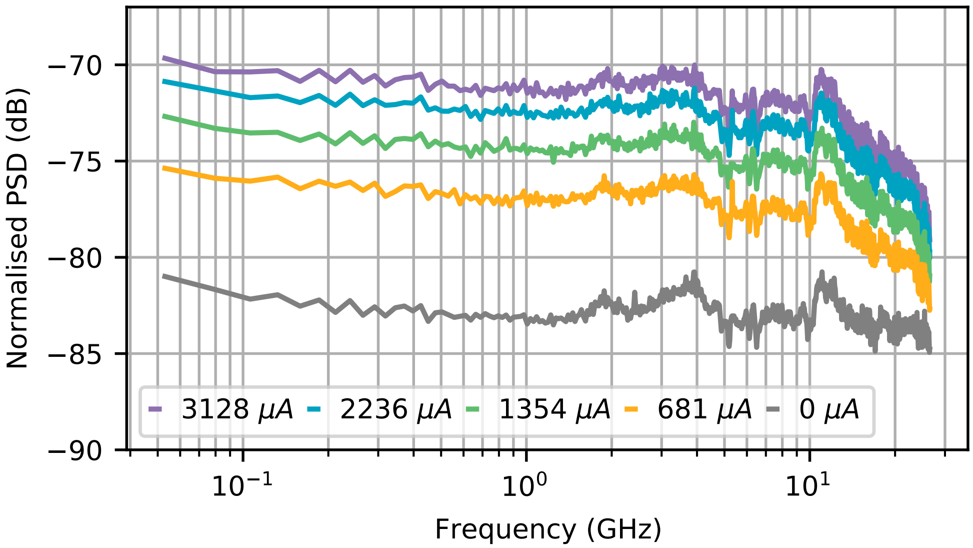

Fig. S3. Raw PSD. This data is directly output from the spectrum analyser used to analyse the performance of the reported detector — this data is presented raw, with zero correction. Eletronic noise corresponds to 0 μA of optical power input to the detector, with legend specifying also other power optical power inputs.

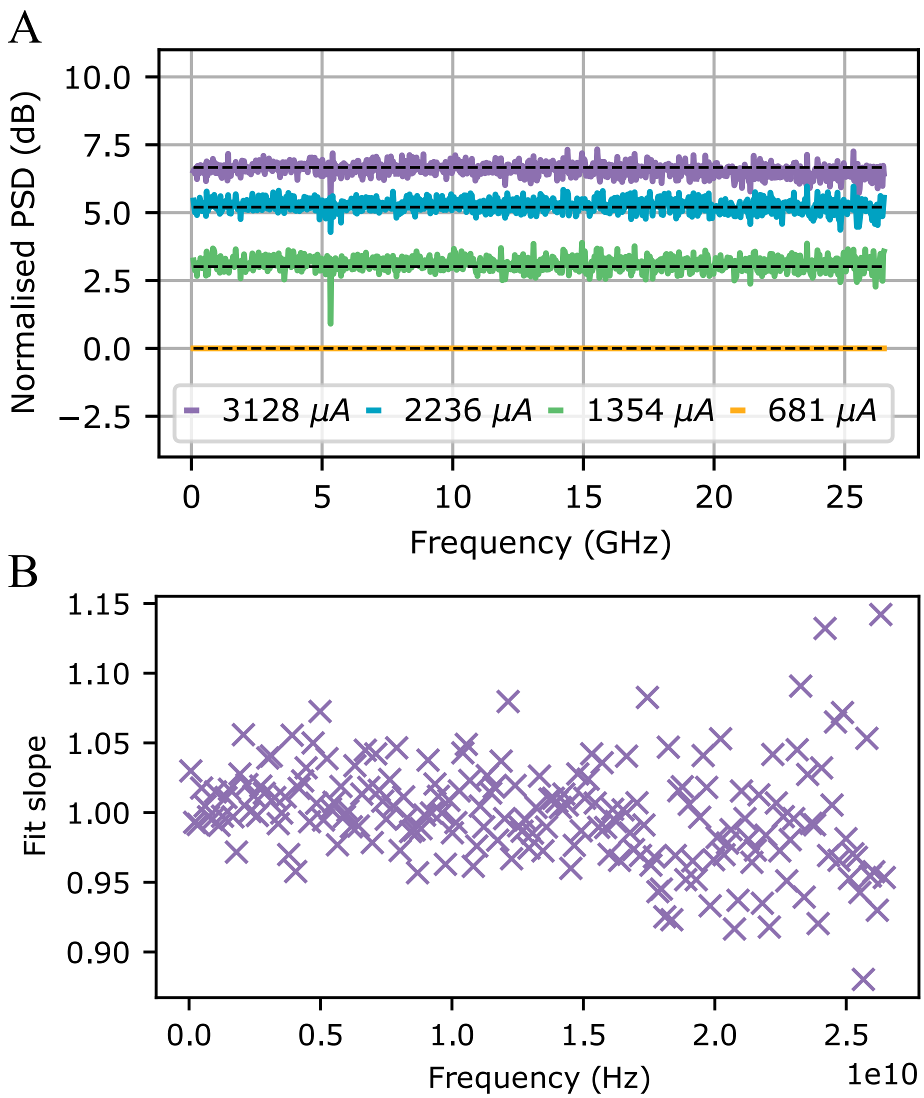

Fig. S4. Linear dependence of PSD on LO power. A, Noise-subtracted PSD traces normalised by the trace of lowest non-zero power. The dashed line overlapping each trace marks the ratio (in dB) between the corresponding photocurrent and the photocurrent measured for the lowest LO power (681 μA). B, Slope of the linear fit of the PSD power dependence at each frequency.
